# The influence of linear and nonlinear pedagogy on motor skill performance: the moderating role of adaptability

**DOI:** 10.3389/fpsyg.2025.1540821

**Published:** 2025-03-31

**Authors:** Qishun Yang, MingLiang Song, Xiao Chen, Min Li, Xiaoan Wang

**Affiliations:** ^1^School of Physical Education, Wuhan Business University, Wuhan, China; ^2^Mass Sports Department, Wuhan Auto Valley Stadium Operation Investment Development Limited Company, Wuhan, China; ^3^School of Sports Training, Tianjin University of Sport, Tianjin, China; ^4^Department of Physical Education, Zhongnan University of Economics and Law, Wuhan, China; ^5^School of General Education, Hunan University of Information Technology, Changsha, China; ^6^School of Physical Education and Health, Hubei Business College, Wuhan, China

**Keywords:** ecological dynamics, constraints-led approach, motor skill acquisition, adaptability, physical education, nonlinear pedagogy

## Abstract

This study addresses the gap in understanding the moderating role of adaptability in the effects of linear pedagogy (LP) and nonlinear pedagogy (NLP) on motor skill performance (MSP), particularly in dynamic sports environments. Recognizing the limited exploration of adaptability’s moderating role in existing literature, this research investigates how adaptability influences the effectiveness of these pedagogical strategies among college students. Forty university males students with no prior soccer experience were randomly assigned to LP or NLP groups and underwent an 8-week training program. MSP was measured by passing successful rate, and adaptability was measured as a composite indicator integrating both the variability of passing types (Variational Pass Rate) and the effectiveness of these variations (Variational Pass Success Rate). Assessments were conducted at three stages: pre-test, post-test, and a 4-week later retention-test. Results demonstrated NLP’s dual superiority: enhancing immediate passing successful rate (post-test *Δ* = 0.09 ± 0.045 vs. LP’s 0.06 ± 0.035, *p* = 0.024) and sustaining skill retention (4-week decline: NLP Δ = -0.013 ± 0.033 vs. LP Δ = −0.032 ± 0.028, *p* = 0.048). Crucially, adaptability significantly moderated NLP’s effectiveness during skill acquisition (pre to post: *β* = 0.478, R^2^ = 0.229, *p* = 0.033), explaining 22.9% of performance variance. This study not only underscores the superior benefits of NLP in fostering enduring motor skills but also highlights the pivotal role of adaptability in tailoring pedagogical approaches. These insights advocate for integrating adaptability assessments into sports education curricula to optimize training outcomes based on individual adaptability levels.

## Introduction

Motor skill performance (MSP), defined as the effective execution of context-specific movement patterns under competitive constraints, is the cornerstone of athletic success in dynamic invasion sports like football (Davids et al., 2005; Aquino et al., 2016). This crucial ability allows athletes to integrate technical precision with tactical awareness, while adapting to three-dimensional challenges: rapidly changing spatial configurations (opponent positioning), temporal demands (game pace), and psychological pressures (performance anxiety) (Lam et al., 2009a, 2009b; Ruan et al., 2022; Ellis and Ward, 2022). However, traditional training methods often lead to a “practice-to-competition gap,” where athletes excel in structured drills but struggle to transfer their skills to match situations that require adaptive responses (Chow et al., 2019). This performance paradox reveals fundamental limitations within conventional linear pedagogy (LP) approaches.

The LP framework employs reductionist skill decomposition and repetitive part-task practice (Kelso, 1995; Schmidt et al., 2018). While effective for initial skill mapping in closed environments, its prescriptive nature creates three critical limitations in dynamic sports: (1) over-standardization of movement patterns reduces tactical flexibility, (2) artificial practice contexts limit perceptual attunement to game cues, and (3) instructor-dominated feedback loops inhibit self-regulatory capacity (Chow et al., 2023; Ranganathan and Newell, 2013). These constraints become particularly problematic when athletes encounter the “adaptive trilemma” of competitive environments: simultaneously managing technical execution, tactical decisions, and psychological regulation under time pressure (Seifert et al., 2017). Studies suggest that LP, with its focus on fixed routines, has limit athletes’ ability to adjust to new situations, leading to reduced performance in dynamic environments (Chow et al., 2021a). To address these limitations, nonlinear pedagogy (NLP) has been proposed as an alternative.

NLP, based on ecological dynamics theory, presents an alternative approach that emphasizes the interaction between the individual, the task, and the environment. NLP fosters self-organization by manipulating constraints (e.g., modifying field dimensions, altering player numbers) to enhance adaptive potential. This develops three key competencies: (1) perceptual attunement to action-relevant cues, (2) functional variability in movement patterns, and (3) meta-cognitive awareness of performance adjustments (Davids et al., 2012; Orth et al., 2019). Emerging research suggests that NLP promotes creativity, adaptability, and better decision-making, which are critical for athletes to excel in dynamic environments (Button et al., 2020; Renshaw and Chow, 2019). In particular, NLP promotes skill adaptation (Chow et al., 2021b), enabling athletes to autonomously adjust their actions in response to environmental feedback, leading to stable, adaptive solutions to complex scenarios in competition (Button et al., 2020; Chow et al., 2021b).

The autonomous skill adaptation observed in NLP-trained athletes—the capacity to modify movement patterns (or technique) in response to environmental perturbations (Renshaw et al., 2022)—fundamentally relies on a deeper psychological construct: adaptability. Adaptability refers to “an individual’s ability, skill, disposition, willingness, and/or motivation to change or fit different task, social, or environmental features (Ployhart and Bliese, 2006; Martin et al., 2012). In the context of sports, adaptability is not only a cognitive trait but a dynamic process that involves motor responses, decision-making, and behavioral (technical) flexibility in the face of real-time challenges (Williams et al., 2018). Specifically, this is reflected in the various adaptive solutions (different techniques and strategies) that are used in the game due to the various conditions that constrain it (Schmidt and Lee, 2019). This ability proves particularly crucial in high-pressure scenarios where athletes must maintain peak performance despite rapidly evolving challenges, a phenomenon extensively documented in elite sport research (Schinke et al., 2012; Lee et al., 2014; Kuok et al., 2021). However, existing studies exhibit a potential research gap regarding individual differences. Specifically, varying levels of adaptability may result in divergent responses to pedagogical approaches (Holliman et al., 2019), influencing how athletes engage with and benefit from both LP and NLP methods.

Current evidence suggests that NLP surpasses LP in enhancing soccer motor skill acquisition (Mohammadi Orangi et al., 2021; Chow et al., 2023; Lindsay et al., 2023b). Notably, while Chow et al. (2023) observed promising skill retention effects in the LP group, the retention-test interval was too short (only one week), necessitating further investigation through extended timeframes. Additionally, conventional indicators such as pass counts and ball possession time predominantly reflect tactical involvement or dominance during gameplay, yet they fail to directly assess the technical quality of skill performance. In contrast, Passing Successful Rate, a widely used indicator in soccer, provides a more direct and reliable reflection of players’ technical abilities (Sarmento et al., 2014), particularly in terms of passing accuracy and performance under match conditions (Liu et al., 2015; Rein et al., 2017). It offers a clearer indication of skill execution compared to indicators like passing count or possession time, which may not fully reflect a player’s technical execution or decision-making in dynamic contexts.

Incorporating adaptability as a moderating factor aligns with NLP’s emphasis on discovering adaptive solutions (Lindsay et al., 2023a). Adaptability, reflected in the variety of passing styles and techniques used to achieve specific objectives in this study, provides a clearer understanding of individual differences in teaching outcomes. This is particularly important for skill execution, retention, and learning transfer in complex environments. By emphasizing adaptability, this approach not only enhances our understanding of how different teaching strategies affect learners, but it also provides valuable insights for sports education reform. Furthermore, it lays a strong theoretical foundation for developing personalized teaching strategies tailored to the individual needs of athletes.

In summary, this study will compare the effects of LP and NLP on improving and maintaining MSP in football, with a particular focus on the moderating role of adaptability. Based on existing literature and theoretical background, the following hypotheses are proposed: H1: In complex team sports, NLP is more effective than LP in promoting MSP. H2: NLP exhibits better retention effects than LP. H3: Individual adaptability moderates the relationship between NLP and MSP. H4: Individual adaptability moderates the relationship between LP and MSP.

## Methodology

### Participants

*A priori* sample size estimation for a 2 × 3 repeated measures ANOVA was conducted using G*Power 3.1.9.7. With a significance level of *α* = 0.05 and a moderate effect size (*f* = 0.25) (Serdar et al., 2021), the analysis indicated that a sample size of 36 was necessary to achieve a high power of 0.9. Participants were recruited via an announcement on the university’s public platform, clearly outlining the inclusion criteria: male, no prior professional sports training, no participation in competitive football matches, aged over 18 years, no underlying health conditions, and occasional exercise (2–3 times per week). A total of 41 participants volunteered for the study. The researchers explained the detailed procedures and requirements of the experiment to all volunteers and conducted a physical fitness assessment, excluding one participant who did not pass the screening. The remaining 40 participants were randomly assigned to two groups: 20 in the LP group and 20 in the NLP group, with participants unaware of their group assignments. Additionally, the anthropometric indicators of the two approaches were presented in Table 1. All participants were provided with a detailed explanation of the study’s purpose and signed an informed consent form. This research was approved by the Academic Committee of Hubei Business College (code 2023030012). As a token of appreciation, all participants received a small gift.

**Table 1:** Age and anthropometric indicators of participants in two groups.

	LP (mean ± SD)	NLP (mean ± SD)
Age (years)	21.05 ± 1.31	21 ± 1.26
Weight (kg)	69.55 ± 3.89	70 ± 3.43
Height (cm)	174.75 ± 4.36	175.2 ± 3.47
BMI	22.77 ± 0.84	22.79 ± 0.55

Both coaches hold master’s degrees and possess coaching certifications at the AFC C level or higher, with over five years of experience in football instruction. The study authors provided the coaches with an overview of the study’s objectives and intervention methods, conducting two workshops totaling four hours on the principles of LP and NLP instruction. Prior to the experiment, a reference class was selected for the coaches to conduct their teaching, and evaluations of their instructional methods were used to assign them to the most suitable teaching approaches. Coaches followed a standardized lesson plan provided by the researchers. To ensure the accuracy of the intervention measures, the researchers communicated with the coaches before each training session and supervised them on-site.

### Procedures and design

Participants in this study undergo a 14-week intervention experiment (see Table 2). Specifically, All participants underwent a pre-test (1st week), followed by an 8-week intervention training period, and concluded with a post-test (9th week). Additionally, a retention-test was conducted four weeks after the post-test (14th week), during which all participants were required to refrain from any sports activities.

**Table 2:** Intervention processes.

Week	Intervention lesson	Lesson topic
1	-	Pre-test
2	1	Passing and receiving
2	2	Passing and receiving
3	3	Dribbles
3	4	Dribbles
4	5	Ball control
4	6	Ball control
5	7	Shooting
5	8	Shooting
6	9	1 vs. 1
6	10	1 vs. 1
7	11	Cross
7	12	Cross
8	13	Create space to invade
8	14	Create space to invade
9	15	Stopping the invasion
9	16	Stopping the invasion
10	-	Post-test
14	-	Retention-test

During the pre-test, post-test, and retention-test, all participants engaged in 5-a-side football matches (5 players per team). Each match lasted 40 min, divided into two 20-min halves, with no stoppage time. The playing field measured 40 meters in length and 25 meters in width, with each team comprising one goalkeeper. To ensure the objectivity of the experimental results, participants were assigned to play in the same teams and against the same opponents. If any participant was unable to participate in a match for specific reasons, a suitable substitute was arranged. However, such a situation did not occur during the experiment.

The intervention training occurred twice a week, with each session lasting 90 min. Each session included a 15-min warm-up, 65 min of topic-related training, and a 10-min cool-down. The 65 min of training employed different teaching approaches (LP and NLP). The training themes for both the LP and NLP approaches were consistent (e.g., ball control, dribbling, passing, and receiving) to ensure uniformity in learning content.

The LP and NLP intervention approaches were adapted from prior research (Chow et al., 2023). The LP approach emphasized repetitive practice under standardized conditions, focusing on the provision of explicit instructions by the coach (e.g., running in for a pass, positioning of the support foot, and the area of foot contact when kicking). It guided participants from simple to complex exercises (from decomposed actions to complete movements). Participants practiced predetermined movement patterns, with the coach providing guidance and correction (Schmidt et al., 2018). NLP teaching focused on adjusting constraints to encourage participants to explore adaptive solutions for achieving skill objectives. It adjusted task limitations based on the participant’s level, reduced direct instructions, and employed more analogical teaching approaches (detailed features can be found in Table 3) (Chow et al., 2019; Rudd J. et al., 2021; Teune et al., 2022). Examples of the LP and NLP lesson plans for the first lesson can be found in Supplementary Table 1.

**Table 3:** Intervention characteristics of two approaches.

Characters	LP	NLP
Teaching methods	Coach-directed, sequential instruction of soccer skills (e.g., passing, shooting, ball control)	Player-centered, encouraging players to independently explore various soccer tactics and skills
Progress	Fixed, standardized training steps, such as learning short passes before long passes	Flexible and personalized, adjusting training content based on players’ specific needs
Exercise methods	Repetitive practice, such as repeated passing and shooting until mastery	Diverse training with different scenarios and constraints, emphasizing adaptability and creativity
Objectives	All players reach the same skill level, such as mastering a standard passing technique	Based on individualized goals, encouraging players to find methods suited to their style
Feedback methods	Coach provides specific feedback, such as correcting passing posture	Encourages self-assessment, with game-like scenarios to help players discover adaptive improvements
Task settings	Consistent tasks and environment, such as standard playing fields and rules	Dynamically adjusts tasks and environment, e.g., changing game rules or ball size
Flexibility	Uses fixed tactics or routines, ensuring all players execute the strategy as planned	Encourages players to explore various tactical options to handle game uncertainties
Instruction	Clear, step-by-step instruction on skill breakdowns	Guided questioning, using analogies and situational teaching

### Measures

#### Motor skill performance measure

In this study, motor skills were assessed through individual passing data collected during match conditions. The matches for the pre-test, post-test, and retention-test were recorded from different angles using two cameras (HDR-CX450, Sony, Japan), followed by the coding of passing actions. The passing action recording sheet was developed with reference to relevant literature (Chow et al., 2023; Mohammadi Orangi et al., 2021) and consultations with several experts, including university professors, head coaches of women’s professional teams, and AFC coaching instructors, resulting in the formation of the passing action recording sheet (see Table 4).

**Table 4:** Passing action recording code.

Types	Definition
Dominant foot inside	Player passes the ball with the inside of dominant foot
Dominant foot outside	Player passes the ball with the outside of dominant foot
Dominant foot Chip pass	Player passes the ball with the toe of dominant foot
Dominant foot Heel pass	Player passes the ball with the heel of dominant foot
Non-dominant foot inside	Player passes the ball with the inside of non-dominant foot
Non-dominant foot outside	Player passes the ball with the outside of non-dominant foot
Non-dominant foot Chip pass	Player passes the ball with the toe of non-dominant foot
Non-dominant foot Heel pass	Player passes the ball with the heel of non-dominant foot
Chest pass	Players pass the ball with their chest
Head pass	Players pass the ball with their heads
Other port of the body	Players pass the ball with other parts of their body
Off-ground passes	Any pass that involves off the ground
Successful pass	A player kicks the ball and his teammate receives it
Total passes	Total number of passes

The dependent variable chosen for this study, motor skill performance, is defined as Passing Successful Rate, which is the ratio of successful passes to total passes. Athletes’ passing accuracy typically decreases significantly under high pressure and limited preparation time (Szczepański and McHale, 2016). Therefore, it is reasonable to select passing accuracy as an indicator of MSP to evaluate the effects of LP and NLP approaches (Chow et al., 2023).

The match footage was evaluated by a researcher (33 years old, a technical analyst for a top women’s professional football club for six years) and an experienced football player (46 years old, with 14 years of coaching experience and a background as a professional player). Importantly, neither evaluator participated in the intervention teaching and was unaware of the group assignments. Moreover, the match footage did not include any identifiers regarding group assignments, ensuring the evaluators were blinded to the groups. An inter-rater reliability analysis was conducted on the passing action recording sheets for all participants, showing a high degree of consistency between the two raters (Cohen’s *κ* = 0.85, *p* < 0.001, 95% CI 0.82–0.92). Additionally, to further strengthen the reliability of the data, an intra-rater reliability analysis was performed, where the same evaluator reassessed the data after a certain period. The results indicated that the two assessments were consistent.

#### The technical adaptability

This study was grounded in the ecological dynamics framework (Button et al., 2020), which posits that adaptability arises from the interplay between movement variability and functional effectiveness. To measure this adaptive capacity, we analyzed passing type variability in football, specifically excluding the dominant foot inside pass, which is recognized as a biomechanically stable and tactically universal technique (Levanon and Dapena, 1998; Ren, 2013).

Building upon the methodological approach proposed by Caso and van der Kamp (2020) as well as insights from elite soccer coaches, we removed this “default” option and operationalized the Variational Pass Rate to quantify passing variability under dynamic constraints. The index is calculated as:


$$\begin{array}{l} \mathrm{Variational Pass Rate}=\\ \frac{\mathrm{Number}\;\mathrm{of}\;\mathrm{passes}\;\mathrm{excluding}\;\mathrm{dominant}\;\mathrm{inside}\;\mathrm{foot}\;\mathrm{passes}}{\mathrm{Total number of passes}} \end{array}$$

However, true adaptability involves more than just trying different techniques. It also requires that these alternative passes are effective in achieving task goals (Chow et al., 2021b). Simply put, merely varying passing methods does not ensure successful performance. Therefore, we also measured the Variational Pass Success Rate, which reflects the effectiveness of these varied passes:


$$\begin{array}{l} \mathrm{Variational Pass Success Rate}=\\ \frac{\mathrm{Number}\;\mathrm{of}\;\mathrm{successful}\;\mathrm{variational}\;\mathrm{passes}}{\mathrm{Total number of Variational Pass}\;} \end{array}$$

Finally, to provide a comprehensive measure of adaptive motor behavior, we combined these two metrics into a single composite indicator:


Adaptability=Variational Pass Rate×Variational Pass Success Rate

This composite indicator quantifies an athlete’s ability to explore alternative movement solutions and execute them effectively under dynamic constraints, and it serves as a reliable indicator for assessing adaptive motor behavior.

### Data analysis

1. This study employed SPSS 27.0 to conduct descriptive statistics on the passing actions of the LP and NLP approaches, with data presented as means and standard deviations (SD). Additionally, significant differences between the two approaches at the same stage were examined. Effect sizes were quantified using Cohen’s d with conventional thresholds: 0.2, 0.5, and 0.8 (Cohen, 1988). Statistical significance was determined using *p* = 0.05.
2. A 2 (Teaching Approach: LP vs. NLP) × 3 (Session: pre/post/retention) mixed-design ANOVA was conducted to determine whether NLP was more effective than LP in maintaining MSP in match scenarios. Effect sizes were represented using partial eta squared, with levels defined as 0.01, 0.06, and 0.14 (Cohen, 1988), and the significance level was set at *p* < 0.05. If significant interaction effects were found, subsequent simple effects analyses were performed.
3. The analysis of moderating effects for repeated measures was conducted using Model 2 from the SPSS 27 Memore plugin 2.1 (Montoya, 2019). In the repeated measures moderation analysis, comparisons between pre-test and post-test (pre-post), as well as post-test and retention-test (post-retention), for both the LP and NLP approaches were analyzed, with MSP as the dependent variable, adaptability as the moderating variable. For significant moderation effects, simple slope analyses were conducted at three levels of the moderator: M − 1SD, M, and M + 1SD (Aiken and West, 1991). This approach was prioritized over the Johnson-Neyman technique due to the absence of statistically detectable transition points, indicating persistent directional effects of the independent variable regardless of adaptability levels.

## Results

### Descriptive statistics

Descriptive statistics revealed distinct patterns in pass type distribution between the two pedagogical approaches across training phases (Table 5). Following the 8-week intervention, the NLP group exhibited significantly greater passing diversity, characterized by increased utilization of unconventional techniques (*F* = 3.907, t(1,38) = −8.774, *p* < 0.01, Cohen’s d = −2.78). This technical difference persisted through the 4-week retention phase (*F* = 2.409, t(1,38) = −12.132, *p* < 0.01, Cohen’s d = −3.84).

**Table 5:** Statistics on different types of passes at each stage.

Session	Dominant foot inside	Dominant foot outside	Dominant foot chip pass	Dominant foot Heel pass	Non-dominant foot inside	Non-dominant foot outside	Non-dominant foot chip pass	Non-dominant foot Heel pass	Chest pass	Head pass	Other port of the body	Off-ground passes	Variational pass
Pre_LP_	17.95 ± 4.83	0.55 ± 0.69	3.6 ± 1.05	0.6 ± 1	0.2 ± 0.41	0.05 ± 0.22	0.5 ± 0.51	0.05 ± 0.22	0.05 ± 0.22	0.4 ± 0.5	0.2 ± 0.41	0 ± 0	6.2 ± 1.91
Pre_NLP_	18.6 ± 5.77	0.4 ± 0.6	3.55 ± 1.47	0.45 ± 0.61	0.05 ± 0.22	0.05 ± 0.22	0.65 ± 0.49	0.1 ± 0.31	0 ± 0	0.3 ± 0.57	0.1 ± 0.31	0 ± 0	5.65 ± 1.79
Post_LP_	23.15 ± 4.85*	0.75 ± 0.72	1.5 ± 0.83*	0.8 ± 0.95	0.2 ± 0.41*	0.05 ± 0.22*	0.5 ± 0.51	0.05 ± 0.22*	0.05 ± 0.22*	0.45 ± 0.61*	0.15 ± 0.37	0.75 ± 0.72*	5.25 ± 2.1*
Post_NLP_	18.95 ± 4.31*	1.1 ± 1.07	2.75 ± 0.97*	1.05 ± 0.69	1.35 ± 0.93*	0.35 ± 0.59*	0.8 ± 0.52	0.3 ± 0.47*	1.3 ± 0.57*	1.5 ± 0.51*	0.15 ± 0.37	2 ± 0.8*	12.65 ± 3.13*
Retention_LP_	24.15 ± 4.61*	0.4 ± 0.6*	0.8 ± 0.7*	0.4 ± 0.5*	0.45 ± 0.76*	0 ± 0*	0.2 ± 0.41*	0.05 ± 0.22*	0.05 ± 0.22*	0.3 ± 0.47*	0.1 ± 0.31*	1.05 ± 0.95*	3.8 ± 2.07*
Retention_NLP_	19.05 ± 5.06*	1.05 ± 1*	2.55 ± 0.69*	1.35 ± 0.81*	1.4 ± 0.68*	0.55 ± 0.76*	0.9 ± 0.64*	0.45 ± 0.51*	0.7 ± 0.66*	1.2 ± 0.7*	0.35 ± 0.59*	2.45 ± 0.83*	12.95 ± 2.67*

*p < 0.05, values are mean plus or minus standard deviation.

The significant between-group differences emerged in five critical pass categories: Dominant foot chip pass (post-test: *p* < 0.01, Cohen’s d = −1.39; retention-test: *p* < 0.01, Cohen’s d = −2.53), Non-dominant foot inside pass (post-test: *p* < 0.01, Cohen’s d = −1.6; retention-test: *p* < 0.01, Cohen’s d = −1.32), Chest pass (post-test: *p* < 0.01, Cohen’s d = −2.88; retention-test: *p* < 0.01, Cohen’s d = −1.32), Head pass (post-test: *p* < 0.01, Cohen’s d = −1.87; retention-test: *p* < 0.01, Cohen’s d = −1.52), and Off-ground passes (post-test: *p* < 0.01, Cohen’s d = −1.65; retention-test: *p* < 0.01, Cohen’s d = −1.58).

### Performance for PE pedagogy among three sessions

This study employed a two-factor repeated measures analysis of variance to compare the effects of LP and NLP approaches on students’ MSP. Normality assumptions were validated, and homogeneity of variance was maintained across measurement phases.

**Figure 1:**
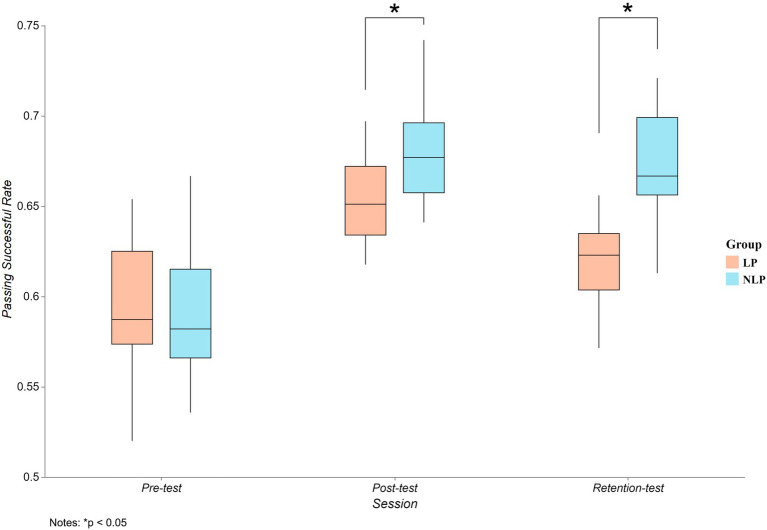
Trend plots of MSP at three different stages of the two approaches.

Figure 1 revealed a significant main effect of teaching approach on MSP (F(1,38) = 11.558, *p* = 0.002, η_p_^2^ = 0.233), with the NLP approach (0.647 ± 0.005) outperforming the LP approach (0.623 ± 0.005). A significant main effect of session was also observed (F(2,37) = 69.176, *p* < 0.001, η_p_^2^ = 0.789). Post-hoc tests indicated that MSP was highest at post-test (0.667 ± 0.004), declined significantly after 4 weeks of detraining (retention-test = 0.645 ± 0.005, *p* < 0.001), but retention-test remained significantly elevated compared to pre-test (0.593 ± 0.005, *p* < 0.001). Furthermore, a significant interaction effect between teaching approach and session was observed (F(2,37) = 5.82, *p* = 0.006, ηp2 = 0.239). These findings suggest that both teaching approach and session significantly contributed to improvements in MSP, warranting further analysis of their interaction effects.

Simple effects analysis for the teaching approach revealed significant differences in MSP across sessions within each teaching approach. Specifically, the LP approach showed significant differences in MSP across sessions (F(2,37) = 27.205, *p* < 0.001, η_p_^2^ = 0.595). Post-hoc comparisons revealed significant improvements from pre-test to post-test (*Δ* = 0.06 ± 0.035, *p* < 0.001, 95% CI [0.037, 0.082]) and from pre-test to retention-test (Δ = 0.027 ± 0.042, *p* = 0.035, 95% CI [0.002, 0.053]). Additionally, the retention-test exhibited significant decrease compared to post-test (Δ = −0.032 ± 0.028, *p* < 0.001, 95% CI [−0.05, −0.015]). The NLP approach also demonstrated significant differences across sessions (F(2,37) = 47.79, *p* < 0.001, η_p_^2^ = 0.721), with significant improvements from pre-test to post-test (Δ = 0.09 ± 0.045, *p* < 0.001, 95% CI [0.067, 0.112]) and from pre-test to retention-test (Δ = 0.077 ± 0.05, *p* < 0.001, 95% CI [0.051, 0.103]). However, the effect of decline from post-test to retention-test was not significant (Δ = −0.013 ± 0.033, *p* = 0.225, 95% CI [−0.03, 0.005]). These findings indicate that after 8 weeks of training, both approaches improved students’ MSP, but on a retention-test after 4 weeks, the MSP was not significantly declined in the NLP approach, whereas it was significantly lower in the LP approach.

Simple effects analysis for sessions revealed no significant differences between two approaches during pre-test (F(1,38) = 0.057, *p* = 0.813, η_p_^2^ = 0.001, 95% CI [−0.019, 0.025]). However, NLP significantly surpassed LP at post-test (F(1,38) = 10.001, Δ = 0.028 ± 0.009, *p* = 0.003, η_p_^2^ = 0.208, 95% CI [−0.045, −0.01]) and retention-test (F(1,38) = 19.78, Δ = 0.047 ± 0.011, *p* < 0.001, ηp2 = 0.342, 95% CI [−0.069, −0.026]), reflecting that students showed no differences at the initial session but exhibited distinct patterns after 8 weeks of training. Specifically, the NLP approach not only improved students’ MSP more effectively but also resulted in more durable effects compared to the LP approach.

Furthermore, the comparison of cross-session improvements highlights the superior performance of the NLP approach in terms of improvement from pre-post (LP = 0.06 ± 0.035, NLP = 0.09 ± 0.045, t(1, 38) = 2.353, *p* = 0.024, Cohen’s d = 0.744) compared to the LP approach. The NLP approach also showed a smaller decline during the retention phase (LP = −0.032 ± 0.028, NLP = −0.013 ± 0.033, t(1, 38) = 2.045, *p* = 0.048, Cohen’s d = 0.647), further supporting its superior effectiveness in maintaining MSP.

### The moderating effect analysis of adaptability

Table 6 showed the relationship between adaptability and MSP across different sessions (pre, post, retention). For the LP approach, adaptability did not significantly predict MSP. However, for the NLP approach, adaptability showed a significant correlation with MSP during both the post-test (R^2^ = 0.351, *β* = 0.592, t(1,18) = 3.12, *p* = 0.006, 95% CI [0.133, 0.681]) and retention-test (R^2^ = 0.243, β = 0.492, t(1,18) = 2.4, *p* = 0.027, 95% CI [0.056, 0.845]). These results indicate that in NLP approach, adaptability demonstrates a strong relationship with MSP. Consequently, we will further explore its moderating effect.

**Table 6:** The relationship between adaptability and MSP.

Approach	Session	R^2^	β	t	p	LLCI	ULCI
LP	Pre-test	0.007	−0.081	−0.346	0.734	−0.848	0.608
	Post-test	0.008	0.087	0.371	0.715	−0.452	0.646
	Retention-test	0.058	0.241	1.052	0.307	−0.279	0.839
NLP	Pre-test	0.012	−0.111	−0.473	0.642	−0.492	0.311
	Post-test	0.351	0.592	3.12	0.006	0.133	0.681
	Retention-test	0.243	0.492	2.4	0.027	0.056	0.845

**Figure 2:**
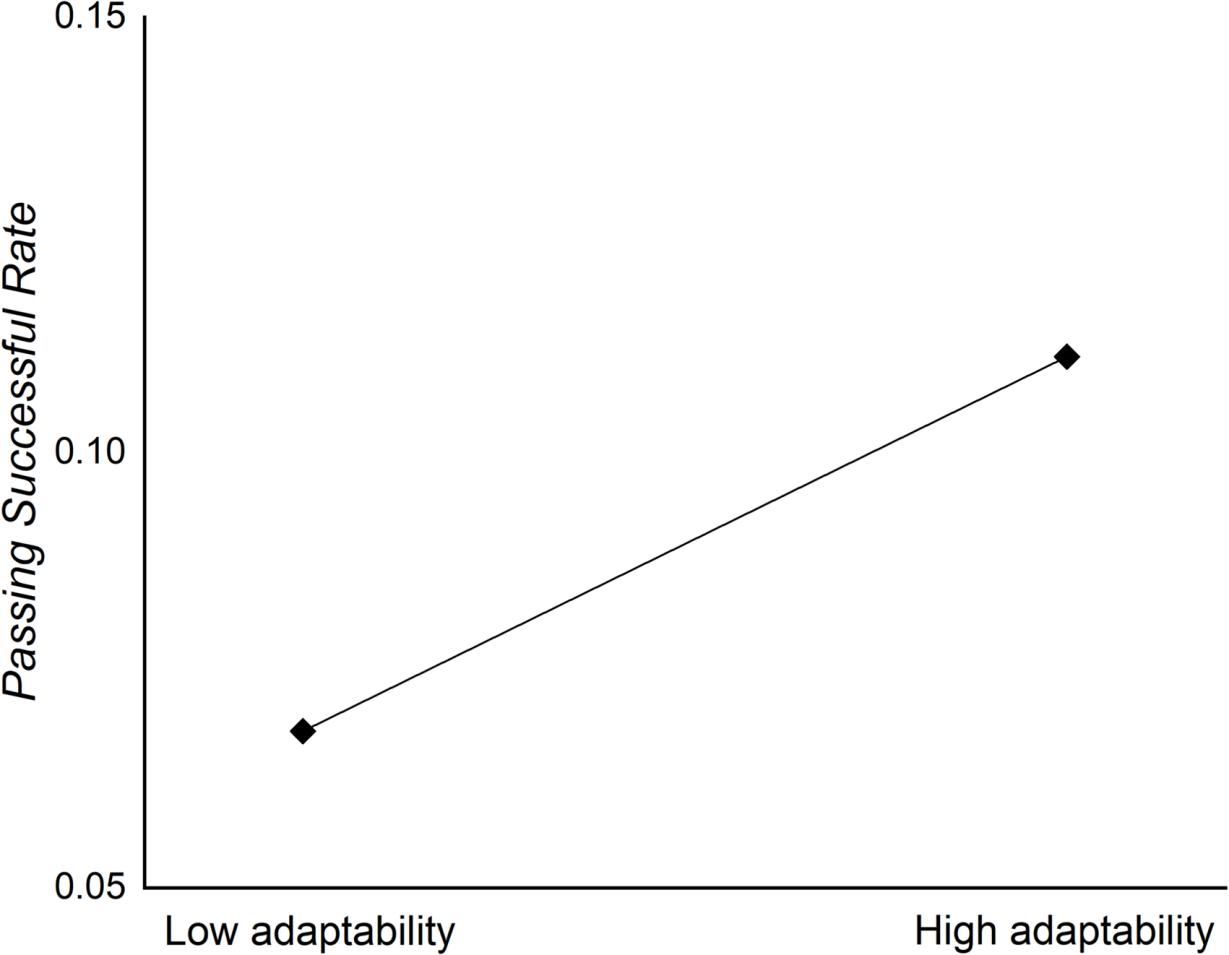
Moderating effect of adaptability on NLP and MSP from pre-test to post-test.

We conducted additional analysis to investigate whether adaptability moderates the relationship between teaching approaches (LP and NLP) and MSP across different sessions (pre-post, post-retention). The results indicated that adaptability did not moderate this relationship for the LP approach across different sessions (pre-post: R^2^ = 0.019, β = 0.139, t(1,18) = 0.594, *p* = 0.56, 95% CI [−0.551, 984]; post-retention: R^2^ = 0.022, β = 0.148, t(1,18) = 0.633, *p* = 0.535, 95% CI [−0.424, 0.79]). For the NLP approach, adaptability did not significantly moderate the relationship during the post-retention session (R^2^ = 0.003, β = 0.057, t(1,18) = 0.244, *p* = 0.81, 95% CI [−0.334, 0.421]). However, adaptability significantly moderated the relationship during the pre-post session (R^2^ = 0.229, β = 0.478, t(1,18) = 2.311, *p* = 0.033, 95% CI [0.045, 0.949]). Furthermore, the simple slope test revealed that High adaptability (B = 0.111, SE = 0.013, *p* < 0.001) exhibited higher MSP than medium (B = 0.09, SE = 0.009, *p* < 0.001) and the low (B = 0.068, SE = 0.013, *p* < 0.001) (see Figure 2).

## Discussion

This study examined the differential effects of LP and NLP on the performance of soccer skills and investigated the moderating role of adaptability. Results indicate that, in the dynamic and unpredictable context of soccer, NLP not only effectively enhances students’ immediate skill performance (supporting H1) but also demonstrates superior skill retention effects (supporting H2). These findings hold significant theoretical and practical implications for motor skill learning and instructional design. Additionally, individual adaptability significantly moderates the effectiveness of NLP (supporting H3), with highly adaptable students showing notable advantages in skill adaptation and passing successful rate. In contrast, adaptability did not significantly moderate the effects of LP (rejecting H4).

### Variability of skill acquisition

The findings further confirmed that NLP fostered greater creative behavior (Santos et al., 2016; Orth et al., 2019; Mohammadi Orangi et al., 2021). The NLP group exhibited significantly more variability in passing techniques. This diversity persisted through the retention phase, underscoring NLP’s capacity to facilitate adaptation to complex game conditions (Button et al., 2020). These findings are consistent with previous studies suggesting that NLP promotes a greater variety of movement solutions, which enhances learner’s ability to adapt to changing demands (Lee et al., 2014; Mohammadi Orangi et al., 2021).

### Advantage in the NLP approach

Due to its emphasis on exploratory learning and variability, NLP promotes skill retention and the ability to effectively find adaptive skills in real-game scenarios (Davids et al., 2007; Ranganathan and Newell, 2013). The results indicated that NLP was superior to LP in improving and maintaining MSP, aligning with existing literature (Chow et al., 2021b; Crotti et al., 2021). From an ecological dynamics systems perspective, NLP facilitated self-organization in students, producing adaptive behavior by adjusting the constraints between the individual, task, and environment (Rudd J. R. et al., 2021). The dynamic systems framework promotes exploratory learning through strategic variability, where learners engage in continuous cycles of search and adaptation. Modifications in passing techniques (e.g., Chip or Off-ground passes) and decision-making approaches (e.g., risk-taking vs. conservative plays) co-adapt to the demands of the environment (Balagué et al., 2019; Chow et al., 2023; Rudd J. R. et al., 2021). This is particularly evident in sports like football and basketball, where athletes undergoing such training develop scenario-specific adaptability, allowing them to adjust pass velocity and angles in real-time when faced with unpredictable defensive constrains (Liu, 2023).

Additionally, contrary to Chow et al.’s (2023) findings under a one-week interval, our four-week interval paradigm reveals decay in the LP group from post-test to retention-test. This decay aligns with schema theory’s prediction of rapid skill erosion without environmental reinforcement (Schmidt et al., 2018). With three weeks of training cessation, students’ initially developed motor schema began to decay due to the lack of continued practice, weakening skill memory pathways and leading to the regression of motor patterns (Savion-Lemieux and Penhune, 2005).

### The role of adaptability

Adaptability emerged as a pivotal moderator of NLP effectiveness, particularly during the pre-test to post-test phase, where it strongly predicted MSP. This finding aligns with the ecological dynamics proposition that adaptable learners reconfigure movement solutions by exploiting functional variability, such as shifting from dominant-foot chip passes to off-ground passes, to meet evolving task constraints (Seifert et al., 2017; Button et al., 2020). In our framework, adaptability encompasses two interdependent processes:

Exploratory flexibility (variability): The capacity to detect and utilize affordances (e.g., selecting weaker-foot passes when defensive pressure limits dominant-foot options).

Functional calibration (effectiveness): The ability to refine exploratory attempts into contextually effective actions (e.g., adjusting pass trajectory to bypass defenders).

The alignment with cross-domain adaptability research (Martin et al., 2013; Holliman et al., 2022) further underscores a universal adaptive principle: individuals who balance exploration and functional precision thrive in unpredictable environments. Within the framework of NLP pedagogy, where coaches deliberately withhold explicit instruction, high-adaptability learners are able to self-organize effective solutions by leveraging movement variability as a functional tool, while low-adaptability learners remain stuck in inflexible, habitual movement patterns. This further underscores the critical role adaptability plays in navigating the complex challenges presented in dynamic sports environments.

However, in the post-test to retention-test phase, no significant moderating effect of adaptability was observed. The diminished moderating effect suggests a shift from exploratory adaptation to functional persistence. During early learning (pre-test to post-test), adaptability facilitates adaptive specialization by enabling players to explore and refine techniques that satisfy individual-environment constraints (e.g., a player’s anthropometrics, opponent positioning) (Renshaw and Chow, 2019). At retention (post-test to retention-test), the focus transitions to reproducing context-tested solutions (Schmidt and Lee, 2019), where previously acquired functional patterns (e.g., off-ground passes calibrated to defensive gaps) must be reliably redeployed despite perturbations (e.g., varying opponent pressure).

### Practical applications

The findings of this study have several practical implications for sports instruction: First, tailoring training programs to individual adaptability levels can optimize learning outcomes. Athletes with higher adaptability thrive in NLP’s exploratory approach, which emphasizes variability and decision-making. Conversely, less adaptable athletes may benefit from a more structured, step-by-step method. For these athletes, coaches should provide additional support through clear guidance and specific feedback to help them gradually adapt to the NLP approach. This may include breaking down tasks into smaller components and offering targeted strategies to help them build confidence and adaptability over time. Second, coaches can design exercises that simulate real-game scenarios, where athletes must adjust to changing conditions, such as opponent positioning or unpredictable game situations. This not only improves technical skills but also fosters better decision-making, helping athletes adapt and perform effectively under pressure. Third, incorporating variability into training sessions can significantly enhance skill retention over time. Its purpose is not only to develop specific skills but also to expose athletes to various changing scenarios, which contributes to the long-term retention of motor skills. Fourth, Training programs should focus on enhancing athletes’ adaptability by challenging them to adjust quickly and apply different strategies in dynamic situations. This boosts both performance and resilience.

### Limitations

This study has several limitations. First, the sample was limited to Chinese male university students, which restricts the generalizability of the findings. Due to various constraints, female participation in sports such as soccer is less often. Future research should include both male and female participants, and consider exploring ways to increase female involvement in football, basketball, and other dynamic sports training programs. Meanwhile, expanding the sample to include diverse cultural backgrounds and age groups would also enhance the external validity. Second, the intervention lasted for only eight weeks, which may not capture the long-term effects on skill stability. Extending the duration of the intervention could provide a more comprehensive understanding of its lasting impact. Lastly, the study did not consider the role of other psychological factors, such as motivation and self-efficacy, in moderating the effects of teaching approaches. Examining these factors could offer a deeper understanding of the psychological mechanisms behind motor skill learning and retention.

## Conclusion

This study confirms NLP’s superiority over LP in enhancing motor skill performance, retention, and skill adaption within dynamic environments. By cultivating adaptive movement solutions (e.g., pass-type diversification through constraint manipulation), NLP bridges the critical gap between structured training and the unpredictability of competitive environments. Crucially, individual adaptability determines NLP’s efficacy: learners with higher adaptability thresholds excel in exploratory training, while others benefit from LP’s structured foundations. These findings highlight the need for adaptability-informed coaching frameworks: NLP for adaptable athletes and LP for skill consolidation. Future work should extend this adaptability-centered paradigm to diverse populations, refining personalized training protocols that align ecological task design with athletes’ neurocognitive profiles.

## Data Availability

The raw data supporting the conclusions of this article will be made available by the authors, without undue reservation.
