# Supplementary material for: The influence of linear and nonlinear pedagogy on motor skill performance: the moderating role of adaptability
Source: Front Psychol. 2025 Mar 31;16:1540821. doi: 10.3389/fpsyg.2025.1540821 (PMC11994730; doi:10.3389/fpsyg.2025.1540821)
Supplement: Supplementary file 1 [file Table_1.DOCX]

# Supplementary Table 1

| **Lesson time: 90 min** | **Linear pedagogy – Lesson 1 Ball Control**  **Number of students: 20**  **Objective： Improve individual player's ball control** | | | |
| --- | --- | --- | --- | --- |
| **Time** | **Activity** | **Instructions** | **Organization** | **Pedagogical Principles** |
| 15 | **Warm-up**   - Jogging - Dynamic stretching |  | Warm up in two lines at the same time |  |
| 20 | **Basic control skill training**  Touch the ball with   - the inside of the foot, - the outside of the foot - the sole of the foot. | - Pay attention to the different parts of the ball that are touched. - Gentle, steady movements | Alternate exercises in pairs | - From slow to fast - From standing exercises to moving exercises - Repeated practice |
| 20 | **Control the ball through obstacles**  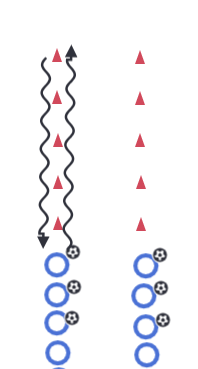 | - Stay close to the football, don't let the ball get out of your control - Touch the back of the ball to bring it forward and touch the sides of the ball to change its direction! | - 12 metres in total length, 3 metres between each cone - Exercise in two lines at the same time - One by one | - From slow to fast - Repeated practice |
| 25 | **1vs1 training**  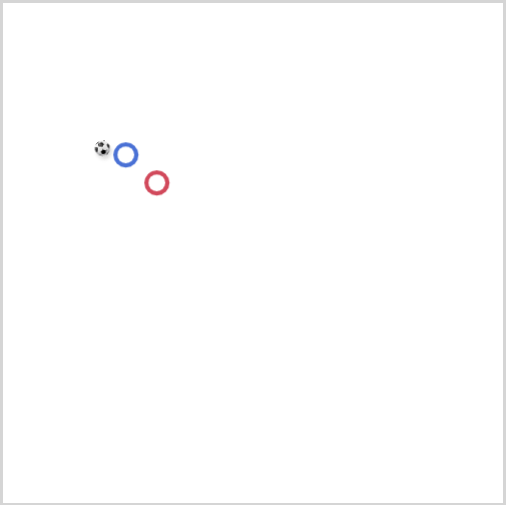 | - Use your body to separate your opponent from the ball - Lower your centre of gravity when protecting the ball - Change the position of the ball | - 1 vs 1 on an 8 metre x 8 metre area. - 10 groups at the same time. | - Building of movement representation - Fixed-assignment context |
| 10 | **Relaxing and Summary**   - Static stretching - Training summary | - Review of teaching and learning - Pointing out the problems | Gather in a group |  |

| **Lesson time: 90 min** | **Nonlinear pedagogy – Lesson 1 Ball Control**  **Number of students: 20**  **Objective： Solve the how to prevent the ball from being taken away by the opponents** | | | |
| --- | --- | --- | --- | --- |
| **Time** | **Activity** | **Instructions** | **Organization** | **Pedagogical Principles** |
| 15 | **Warm-up**   - Jogging - Dynamic stretching |  | Warm up in two lines at the same time |  |
| 20 | **Dodging**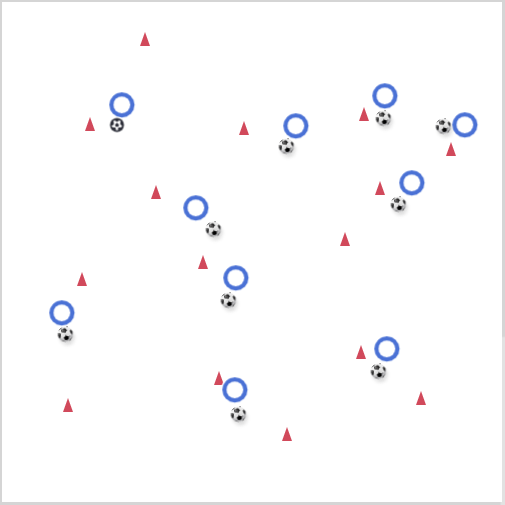 | - No specific ball control skill requirements were given - Don't hit cones and other players - Look up and observe the surroundings | - 10 players free with the ball in an area of 15 metres x 15 metres - As the practice progresses, the coach enters the square and begins to throw the marker. Players dodge the marker thrown by the coaches, as well as, cones and other players. | - Setting constraints (e.g. no touching the ball with the inside of the foot, outside of the foot, etc.) - Vary tasks by student status - Change the size of the field - Change dribbling speed in response to coaches' signals |
| 20 | **Protect the ball**  **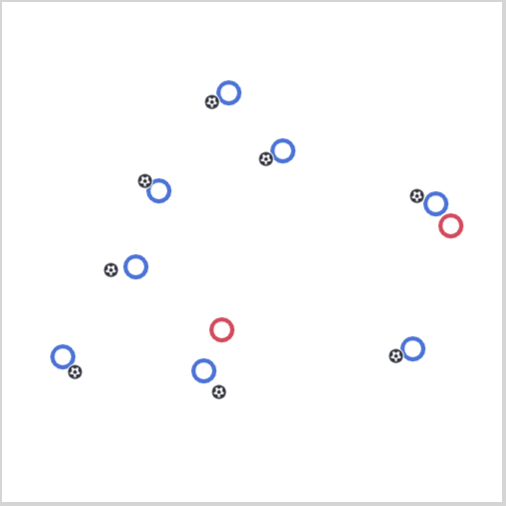** | - No specific ball control skill requirements were given - Look up and observe the surroundings - Losing the ball and getting it back quickly | - 8 player free with the ball in an area of 15 metres x 15 metres, as well as, 2 players tackling the ball. - After 2 minutes the player without the ball does 10 push-ups. | - Setting constraints (e.g. no touching the ball with the inside of the foot, outside of the foot, etc.) - Change the size of the field - Increase and decrease in the number of defence players |
| 25 | **2vs2 training**  **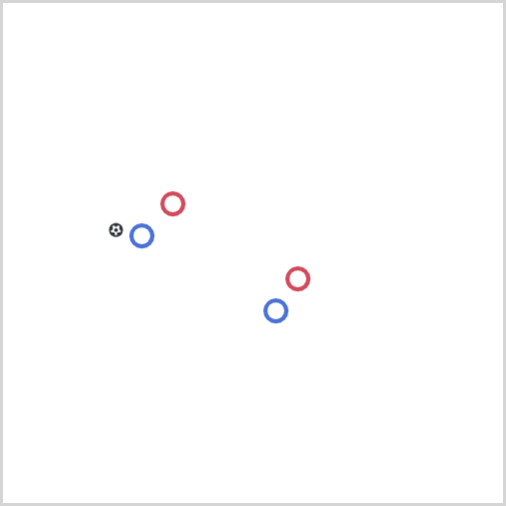** | - Use all methods to keep the ball in the team's possession | - 2 vs 2 on an 12 metre x 12 metre area.   10 groups at the same time. | - Setting constraints (e.g. no touching the ball with the inside of the foot, outside of the foot, etc.) - Change the size of the field - Encourage player to explore the many different ways |
| 10 | **Relaxing and Summary**   - Static stretching - Training summary | - Review of teaching and learning - Pointing out the problems | Gather in a group |  |
